# Thoracic Erector Spinae Plane Catheter as a Bridge to Patient-Controlled Thoracic Epidural Analgesia in Unilateral Lung Transplantation

**DOI:** 10.7759/cureus.31770

**Published:** 2022-11-22

**Authors:** Jason Podolnick, Cierra Grubbs, Jennie Johnson, Anna Weyand, Roland Flores

**Affiliations:** 1 Anesthesiology, University of Colorado Anschutz Medical Campus, Aurora, USA; 2 Surgery, University of Colorado Anschutz Medical Campus, Aurora, USA; 3 Anesthesiology, US Anesthesia Partners of Colorado, Greenwood Village, USA

**Keywords:** opioid, regional anesthesia, epidural, acute pain management, lung transplant

## Abstract

Erector spinae plane (ESP) blocks may be an acceptable alternative to thoracic epidural analgesia during the postoperative period in lung transplant patients. In this case report, we describe the use of an ESP block to manage acute postoperative pain in a unilateral lung transplant, although it was inferior to the thoracic epidural, which was eventually placed.

## Introduction

Management of acute postoperative pain in lung transplantation can be challenging and is critically important to prevent complications, including ineffective breathing and clearing of secretions, which may impede recovery or lead to reintubation. The necessary thoracotomy frequently results in significant pain from disrupting the ribs, muscles, intercostal nerves, costovertebral joints, and pleura. While multiple options for pain control may be acceptable, thoracic epidural analgesia is significantly superior to intravenous opioids for pain management as well as patient satisfaction for thoracic surgery in general and lung transplantation specifically [1,2]. In addition, thoracic epidural analgesia can help minimize postoperative mechanical ventilation time and reduce opioid consumption [3].

While a preoperative thoracic epidural offers excellent analgesia, the lung transplant procedure can require systemic heparinization for the cardiopulmonary bypass, which may warrant an unacceptable transplant delay after the cannulation of a blood vessel during the placement of the epidural [4]. Therefore, these epidurals are often placed postoperatively, but unfortunately, clotting and platelet abnormalities may continue into the postoperative period and preclude the placement of an epidural at that time as well [5].

It has been suggested that erector spinae plane (ESP) blocks may be an acceptable alternative to thoracic epidural analgesia in the postoperative period in thoracotomy and lung transplant patients [6-8]. In this case report, we describe an instance of an ESP block facilitating effective management of acute postoperative pain in lung transplant and used as a bridge to a thoracic epidural, which was eventually placed.

## Case presentation

**Figure 1:**
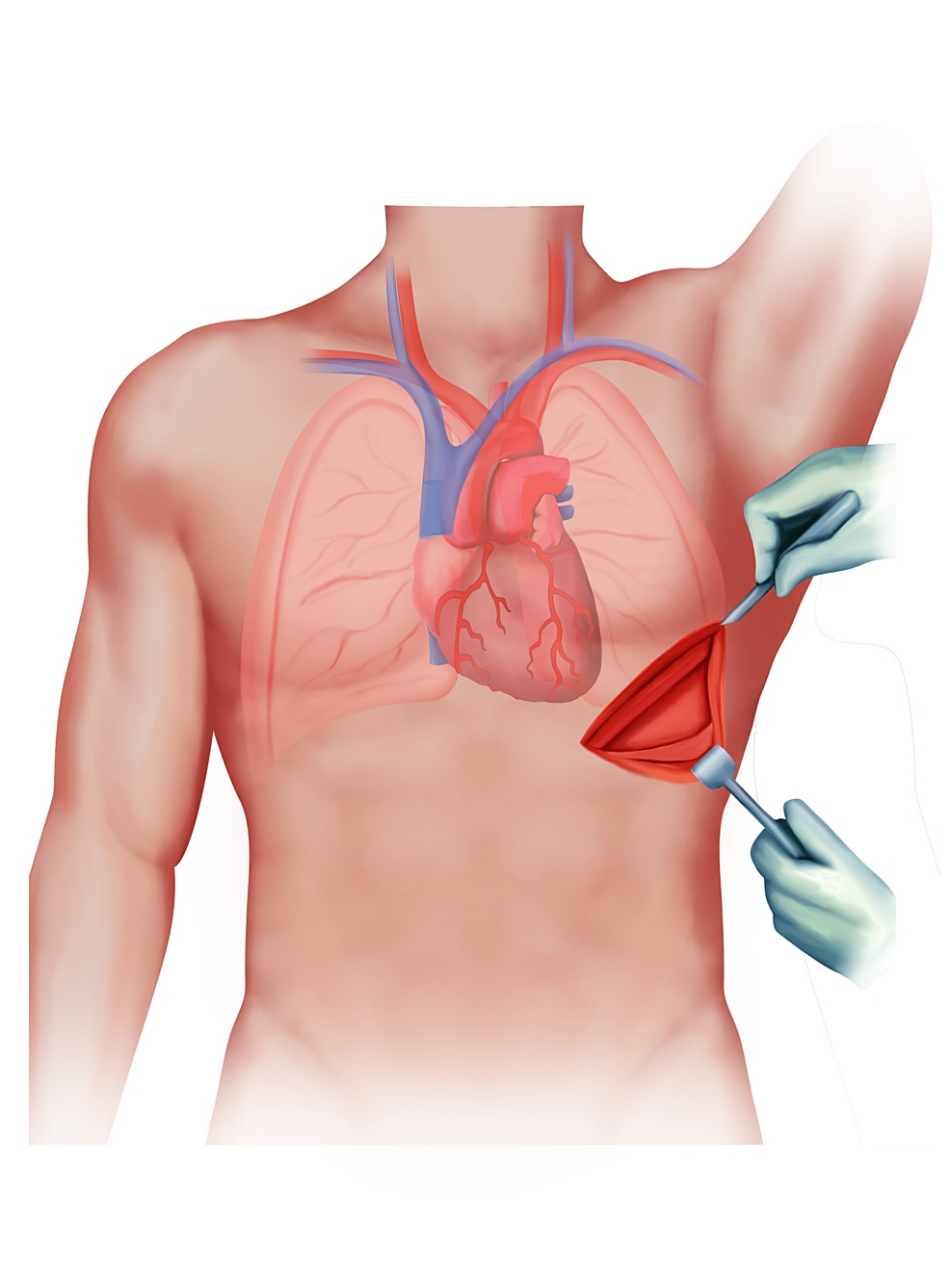
Surgical approach - left anterior thoracotomy at the left fifth intercostal space.

A 68-year-old male patient presented to a tertiary academic referral center for single lung transplantation. Consent was obtained for the procedure and publication from the patient. The patient had a past medical history significant for hypertension, chronic obstructive pulmonary disease, chronic hypoxic respiratory failure, and chronic hypercapnia. The patient’s baseline home oxygen requirement was 6-8 L by nasal cannula. The transplant was performed through a left anterior thoracotomy at the left fifth intercostal space, with the sixth rib shingled posterolaterally, and a cardiopulmonary bypass was utilized during the procedure. The surgical approach is shown in Figure 1.

**Figure 2:**
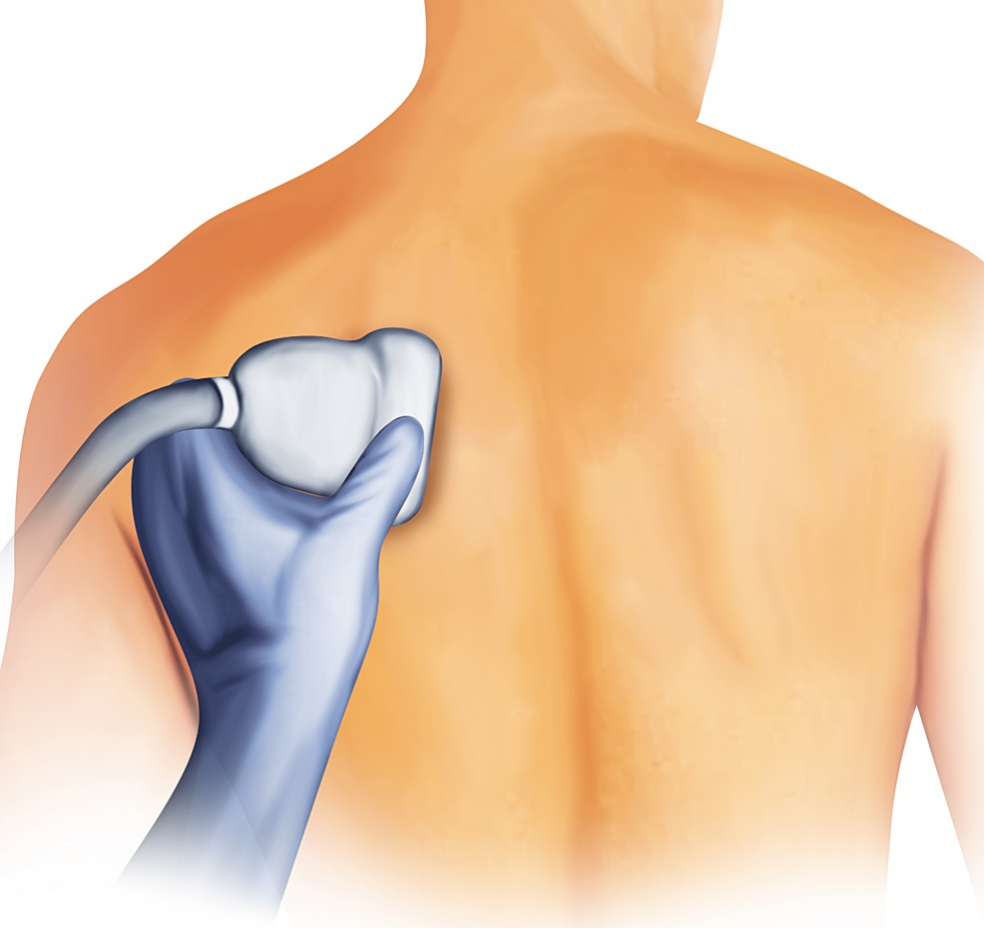
Ultrasound probe placement for the erector spinae plane block. The probe is placed in the parasagittal orientation to the left of the spinous processes to identify the trapezius muscle, rhomboid major muscle, erector spinae muscle, and the transverse process at the T5 level.

**Figure 3:**
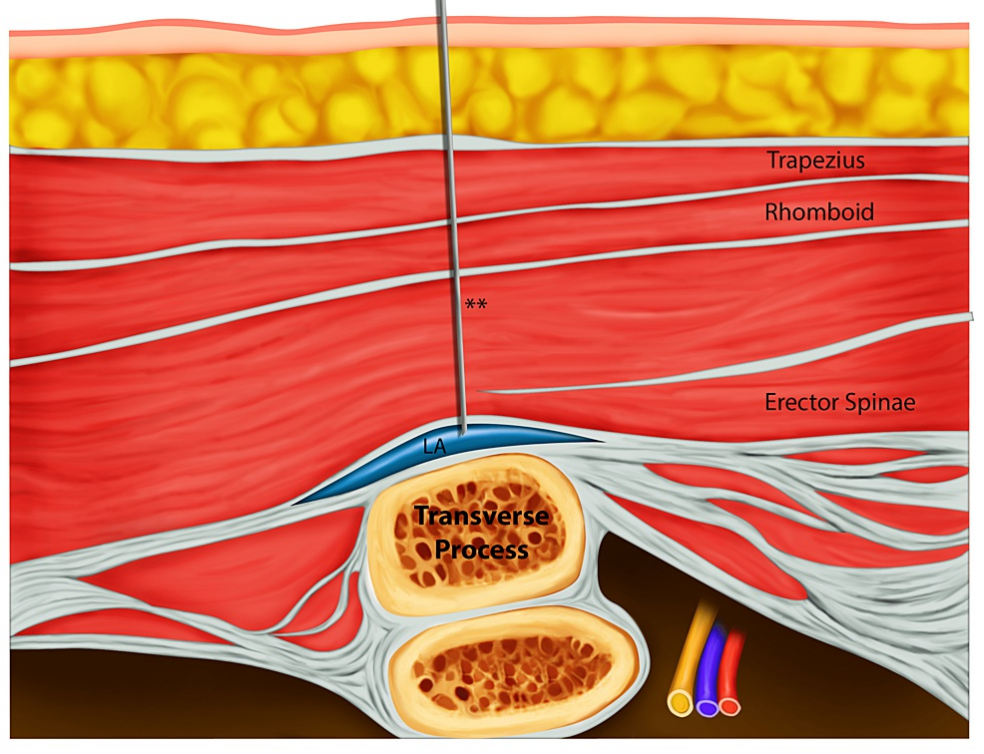
Erector spinae plane block anatomical cross-section showing the out-of-plane approach. Representation of anatomy to be viewed during ultrasound-guided block: needle (**). LA, local anesthetic

The patient was successfully weaned from cardiopulmonary bypass. A left posterior right angle 32-F chest tube and left anterior apical straight 28-F chest tube were placed. The double-lumen endotracheal tube was exchanged for a single-lumen tube after the case, and the patient was transferred to the cardiothoracic intensive care unit in stable condition. The surgical team and intensivists consulted our hospital’s acute pain service for the placement of a thoracic epidural to manage acute postoperative pain and help prevent reintubation. During the consultation, it was discovered that the patient had an international normalized ratio (INR) of 1.5, which precluded the placement of an epidural based on the American Society of Regional Anesthesia (ASRA) Pain Medicine coagulopathy guidelines [9]. On postoperative day 1, in place of a thoracic epidural, our service instead placed an ultrasound-guided, left-sided ESP catheter as there are case reports showing this technique to be safe in patients with altered hemostasis [10]. The intensivists extubated the patient to a heated high-flow nasal cannula before the initiation of the ESP catheter procedure. The ESP catheter was placed on the left side at the T5 level. Figure 2 shows the placement of the ultrasound probe, and Figure 3 shows the anatomy that will be seen by ultrasound as well as an out-of-plane trajectory of the block needle.

We initiated the block with a bolus of 30 mL of 0.5% bupivacaine. After the initial block and catheter placement, 0.2% ropivacaine was infused at 1 mL/hour to keep the catheter patent and bolused through the pump with 20 mL every four hours. Initially, the patient’s pain scores decreased from 7/10 to 3/10 after catheter placement. In the 24 hours following catheter placement, the patient used 15 mg and then another 10 mg of oral oxycodone to supplement his analgesia. The patient was also receiving 975 mg of acetaminophen every eight hours. On postoperative day 3, the patient continued to intermittently utilize heated high-flow oxygen by nasal cannula and bilevel positive airway pressure (BiPAP). The patient stated that while his pain was improved, it would increase to 6/10 before each bolus, and he felt it was inhibiting him from taking deep breaths during these periods of increased pain. As his INR fell to 1.3, the acute pain service team decided to remove the erector spinae catheter and replace it with a T4-5 patient-controlled thoracic epidural analgesia catheter to help improve analgesia and respiratory function. The epidural was infused with 0.0625% bupivacaine with 5 microg/mL of hydromorphone at a basal rate of 6 mL/hour with the ability for the patient to self-bolus 3 mL every 15 minutes. Following the placement of the patient-controlled epidural analgesia catheter, pain scores ranged from 0/10 to 2/10, and no oral or intravenous opioids were utilized. In addition, the patient stopped needing heated high-flow oxygen and BiPAP as 2 L of oxygen by nasal cannula was sufficient after the epidural placement.

## Discussion

It is common for our acute pain service to be consulted to assist with pain management on postoperative day 1 following lung transplantation surgery. The gold standard for pain control in thoracotomy is thoracic epidural placement, which affords excellent analgesia while minimizing complications in lung transplant recipients [1,2]. In addition, the pain relief provided can decrease systemic opioid requirements and improve pulmonary mechanics. However, as was seen in this case presentation, these patients are frequently coagulopathic postoperatively, which can last for several days, precluding epidural placement.

When the placement of a thoracic epidural is not possible, ESP catheters can be utilized to manage postoperative pain after thoracotomy [6,11]. These can be done either unilaterally or bilaterally, depending on the surgery performed. The goal of ESP catheters is to improve pain control and respiratory mechanics while minimizing opioid usage in place of an epidural. One case series of 42 patients demonstrated that when performed at the T5 level, the patients had decreased sensation in a dermatomal distribution from T3 to T10, with no significant adverse effects [6]. While several studies demonstrate ESP blocks' efficacy in minimizing opioids for various thoracic surgeries [6,11], there is very little in the literature about using ESP catheters in lung transplant patients.

The placement of ESP catheters does not risk neuraxial bleeding and, therefore, does not have the same stringent anticoagulation guidelines as the placement of a thoracic epidural. Due to the location of the block and its distance from the spine, it may be considered a low-risk procedure based on ASRA Pain Medicine guidelines [9]. For example, one case series studying the use of ESP catheters for pain control in left ventricular assist device patients demonstrated that ESP blocks could safely be performed in patients receiving therapeutic anticoagulation with no significant adverse events [12]. Our institution has safely placed ESP catheters in anticoagulated patients and those with coagulopathies, including postoperatively in lung transplant patients.

## Conclusions

In this case, an ESP catheter was effective but still inferior to the thoracic epidural. Although our patient’s pain scores decreased after ESP catheter placement and he did not need to be reintubated, he utilized systemic opioids, heated high-flow oxygen, and BiPAP until we placed the thoracic epidural. After epidural placement, pain scores immediately improved, systemic opioid usage decreased to zero, and the patient was able to be weaned from high-flow oxygen and BiPAP to a standard nasal cannula.

This case report shows that an ESP catheter can be an effective technique as part of a multimodal analgesic regimen for lung transplant patients. This can be beneficial in helping improve respiratory mechanics, thereby decreasing postoperative pulmonary complications and decreasing supplemental oxygen requirements after extubation. Furthermore, improved analgesia can reduce opioid usage and help mitigate opioid-related side effects. In this case, while the epidural that was subsequently placed was found to be somewhat superior, the ESP catheter helped keep the patient from being reintubated and served as a safe and effective bridge to the more definitive analgesic modality.
